# Effect of Cryopreservation on Proteins from the Ubiquitous Marine Dinoflagellate *Breviolum* sp. (Family Symbiodiniaceae)

**DOI:** 10.3390/plants10081731

**Published:** 2021-08-21

**Authors:** Hsing-Hui Li, Jia-Lin Lu, Hui-Esther Lo, Sujune Tsai, Chiahsin Lin

**Affiliations:** 1 National Museum of Marine Biology & Aquarium, Pingtung 944, Taiwan; hhli@nmmba.gov.tw (H.-H.L.); estherlo1995@yahoo.ca (H.-E.L.); 2 Institute of Marine Biology, National Dong Hwa University, Pingtung 944, Taiwan; alice0783@yahoo.com.tw; 3 Department of Post Modern Agriculture, Mingdao University, Peetow, Chang Hua 369, Taiwan

**Keywords:** cryopreservation, Symbiodiniaceae, two-step freezing, protein expression, coral

## Abstract

Coral reefs around the world are exposed to thermal stress from climate change, disrupting the delicate symbiosis between the coral host and its symbionts. Cryopreservation is an indispensable tool for the preservation of species, as well as the establishment of a gene bank. However, the development of cryopreservation techniques for application to symbiotic algae is limited, in addition to the scarceness of related studies on the molecular level impacts post-thawing. Hence, it is essential to set up a suitable freezing protocol for coral symbionts, as well as to analyze its cryo-injury at the molecular level. The objective of this study was to develop a suitable protocol for the coral symbiont *Breviolum* subjected to two-step freezing. The thawed *Breviolum* were then cultured for 3, 7, 14, and 28 days before they were analyzed by Western blot for protein expression, light-harvesting protein (LHP), and red fluorescent protein (RFP) and tested by adenosine triphosphate bioassay for cell viability. The results showed the highest cell viability for thawed *Breviolum* that was treated with 2 M propylene glycol (PG) and 2 M methanol (MeOH) and equilibrated with both cryoprotectants for 30 min and 20 min. Both treatment groups demonstrated a significant increase in cell population after 28 days of culture post-thawing, especially for the MeOH treatment group, whose growth rate was twice of the PG treatment group. Regarding protein expression, the total amounts of each type of protein were significantly affected by cryopreservation. After 28 days of culture, the protein expression for the MeOH treatment group showed no significant difference to that of the control group, whereas the protein expression for the PG treatment group showed a significant difference. *Breviolum* that were frozen with MeOH recovered faster upon thawing than those frozen with PG. LHP was positively and RFP was negatively correlated with Symbiodiniaceae viability and so could serve as health-informing biomarkers. This work represents the first time to document it in Symbiodiniaceae, and this study established a suitable protocol for the cryopreservation of *Breviolum* and further refined the current understanding of the impact of low temperature on its protein expression. By gaining further understanding of the use of cryopreservation as a way to conserve Symbiodiniaceae, we hope to make an effort in the remediation and conservation of the coral reef ecosystem and provide additional methods to rescue coral reefs.

## 1. Introduction

Cryopreservation is a technique that prolongs the viability of structurally intact cells and tissues by freezing under low-temperature treatment at relatively low cost and time [1]. Its procedure can also reduce the expense and work required for the culture of algae, as opposed to the traditional method, and prevent possible contamination and genetic shift during culture [2].

In recent years, temperature change, acidification, and eutrophication [3,4] have caused great impacts on the marine environment, leading to catastrophic consequences on the coral reef ecosystems. Coral reefs, the irreplaceable habitats for many marine organisms, are currently endangered by the disruption of their symbiotic relationship with symbiotic dinoflagellates from the Symbiodiniaceae family due to global environmental change. The disruption of the symbiosis ceases the nutrient supply for corals from Symbiodiniaceae photosynthesis, leading to coral bleaching [5,6]. Corals cannot survive without their symbiotic dinoflagellates, and therefore, conserving the symbionts will be a critical factor in coral reef conservation [7].

Much research is now focused on the application of cryopreservation on Symbiodiniaceae conservation [8,9]. For example, Zhao et al. 2017 [10] tested for the optimal cryopreservation protocol of *Gerakladium* extracted from *Gorgonacea*, using vitrification and slow freezing method. In addition, Chong et al. 2016c [11] made the first successful attempt on the two-step freezing of *Gerakladium*, using 1 M MeOH with 0.4 M sucrose as a cryoprotectant (CPA); the survival rate of the thawed Symbiodiniaceae reached 56.93%. By using Symbiodiniaceae extracted from *Pseudopterogorgia elisabethae*, Santiago-Vázquez et al. 2017 [12] found that, of four different freezing protocols, the two-step freezing resulted in the highest survival rate of thawed algae during culture. Furthermore, Hagedorn and Carter, 2015 [13] attempted cryopreservation of Symbiodiniaceae from different seasons of a year and found that *Cladocopium* quality varies with season. The information accumulated through the preceding studies aids in the establishment of a database for the gene bank of symbiotic dinoflagellates.

Aside from symbiotic dinoflagellates, two-step freezing is also being commonly applied to other algae. For example, in a 1984 study conducted by Van Der Meer and Simpson, the rhodophyta *Gracilaria tikvahiae* was preserved for as long as 4 years using the two-step freezing. Zhang et al. 2008 [14] also performed the same procedure on the economically valuable algae *Laminaria japonica*; of the 43% of algae that survived, there was more of the male gametophyte than female gametophyte, suggesting that two-step freezing could possibly assist in selective breeding in the future. The two-step freezing involves two stages of cooling: the cell is first cooled to between −30 and −50 °C [15,16], then it is immersed into liquid nitrogen. The purpose of the first cooling stage is to equilibrate the cellular osmotic pressure with the implementation of cryoprotectants (CPAs) to efflux excess water from the cell, thus preventing the formation of intracellular crystals during cryopreservation [2,17].

Current studies on topics regarding the molecular level impact of cryopreserved Symbiodiniaceae only cover as much as mitochondria DNA [18], gene expression [19,20], and DNA integrity [12]. Most research conducted on the protein expression of Symbiodiniaceae focused on how environmental factors influence it, such as the affected protein expression and gene loss caused by different light intensity [21] and warming of seawater [22], as well as the impact of photosynthesis on Symbiodiniaceae protein level and chlorophyll expression, and on the endosymbiotic relationship between algae and coral [23]. However, relevant research on the impact of protein in cryopreserved Symbiodiniaceae is still lacking. Although the role of red fluorescence proteins (RFP) had been extensively studied, its research is still a highly discussed topic [24,25]. Meanwhile, Huang et al. (2017) [26] had explored a high titer antibody for Symbiodiniaceae light-harvesting protein (LHP), which is involved in photosynthesis [27,28]. The objective of the study is to obtain a suitable protocol for the two-step freezing of *Breviolum*, as well as to observe the growth of post-thawed *Breviolum* in culture. The present study also investigated the effect of cryopreservation at the molecular level in *Breviolum* by analyzing proteins (RFP and LHP).

## 2. Results

### 2.1. Effect of Equilibration Time and Concentration of CPAs on the Viability of Frozen-Thawed Breviolum

**Figure 1:**
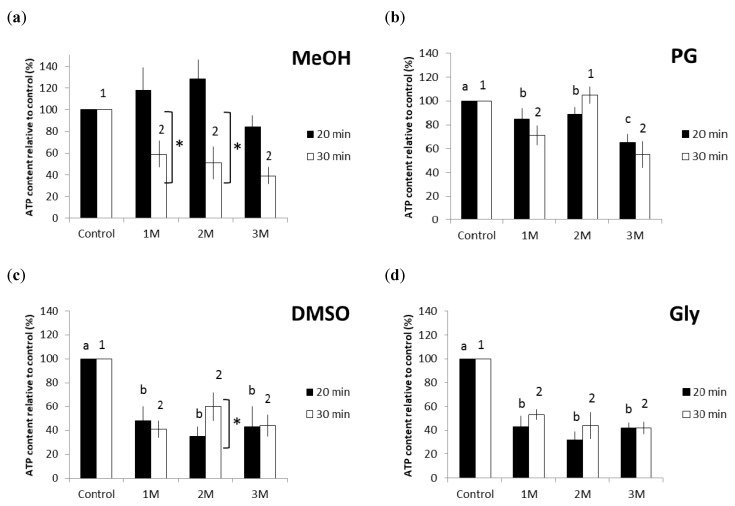
ATP content for thawed *Breviolum* that was cryopreserved with different CPAs: (**a**) MeOH, (**b**) PG, (**c**) DMSO, and (**d**) Gly. *Breviolum* was cryopreserved with 4 different types of CPAs at 3 varying concentrations (1, 2, and 3 M) and equilibrated for either 20 min or 30 min. The control group represents *Breviolum* not subjected to any treatment. Different letters and numbers represent a significant difference (*p* < 0.05) in treatment groups of varying concentrations equilibrated for 20 and 30 min. Asterisks are marked for any significance difference observed under the same CPA concentrations but over different equilibration times. The error bar represents standard deviations.

The experiment investigated the viability of thawed *Breviolum* that were treated with four types of CPAs (MeOH, PG, DMSO, and Gly) at three varying concentrations (1, 2, and 3 M) and equilibrated for either 20 or 30 min (Figure 1). ATP content (presented as relative percentages against experimental controls) was used as a proxy for cell viability. Between the MeOH treatment groups equilibrated for 20 and 30 min, the former showed relatively higher *Breviolum* viability after cryopreservation (Figure 1a). The MeOH treatment group equilibrated for 20 min showed significantly higher *Breviolum* viability in the 1 and 2 M groups than at 30 min (pairwise *t*-tests, *p* = 0.021 and *p* = 0.035, respectively). In the PG treatment group equilibrated for 20 and 30 min, it was found that thawed *Breviolum* treated with 2 M PG and equilibrated for 30 min had the highest cell viability (Figure 1b). Moreover, there were no effects of equilibration time (20 vs. 30 min) at any of the three PG concentrations (Figure 1b). Aside from all that, the results revealed that, in the PG treatment group equilibrated for 20 min, *Breviolum* viability decreased with increasing CPA concentration (one-way ANOVA, F(3/8) = 39.452, *p* < 0.001). Lastly, there was no significant difference (*p* > 0.05) in *Breviolum* viability among the DMSO and Gly treatment groups (Figure 1c,d, respectively), regardless of equilibration time or CPA concentration; the only exception to this was the 2 M DMSO treatment group, which showed a significant temporal difference (pairwise *t*-tests, *p* = 0.029).

The experiment yielded the highest *Breviolum* viability for the 2 M MeOH treatment, equilibrated for 20 min, with an increase in ATP response reaching 130%. The next best symbiont viability was obtained with the 2 M PG group equilibrated for 30 min, with an ATP response reaching 100%. Ensuing were the 2 M DMSO and 1 M Gly treatment groups, both equilibrated for 30 min, at 60% and 58%, respectively. The MeOH and PG treatments yielded higher *Breviolum* viability than either of the DMSO and Gly treatment groups. The Gly treatment group displayed its highest *Breviolum* viability with a 1 M concentration, whereas the other three CPA treatment groups showed their respective highest viability with a 2 M concentration. In terms of equilibration time, only the MeOH treatment group displayed its highest *Breviolum* viability with 20 min, whereas with the other three CPAs, the 30 min equilibration yielded the best *Breviolum* viability.

### 2.2. Breviolum Growth Rate after Four Culture Periods

**Figure 2:**
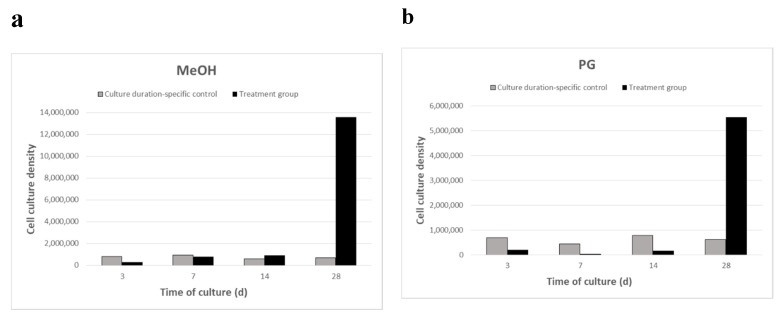
Cell culture density for the MeOH (**a**) and PG (**b**) treatment groups of thawed *Breviolum* after 3, 7, 14, and 28 days of culture; each culture duration trial was conducted independently. Control densities for the MeOH treatment: 3.16 × 10^5^ (3-day culture), 8.16 × 10^5^ (7-day culture), 9.27 × 10^5^ (14-day culture), and 1.36 × 10^7^ cells/mL (28-day culture); PG treatment: 2.00 × 10^4^ (3-day culture), 3.20 × 10^4^ (7-day culture), 1.67 × 10^5^ (14-day culture), and 5.54 × 10^6^ cells/mL (28-day culture).The horizontal axis shows the number of days of culture, at which 0 days represents the cell population immediately after thawing.

Thawed *Breviolum* were cultured independently under different culture durations (3, 7, 14, and 28 days), during which growth was quantified (Figure 2). The number of algal cells was determined using a hemocytometer. The cell culture density for the control groups (enumerated immediately post-thaw) for the MeOH group *Breviolum* was 8.28 × 10^5^ (3-day culture), 9.35 × 10^5^ (7-day culture), 6.16 × 10^5^ (14-day culture), and 7.10 × 10^5^ cells/mL (28-day culture) (Figure 2a). The cell culture density for the control groups for the PG group *Breviolum* was 6.85 × 10^5^ (3-day culture), 4.36 × 10^5^ (7-day culture), 7.87 × 10^5^ (14-day culture), and 6.27 × 10^5^ cells/mL (28-day culture) (Figure 2b). Results showed that *Breviolum* growth commenced after 7 days of culture for the MeOH treatment group (Figure 2a) and after 14 days of culture for the PG treatment group (Figure 2b). Both treatment groups displayed growth by the 28th day of culture, but the cell culture density of the MeOH treatment group was twice as high as that of the PG treatment group. The cell culture density and normalized percentage for MeOH group *Breviolum* after 3, 7, 14, and 28 days of culture were 3.16 × 10^5^, 8.16 × 10^5^, 9.27 × 10^5^, and 1.36 × 10^7^ cells/mL, respectively (Figure 2a), whereas that of PG group *Breviolum* were 2.00 × 10^4^, 3.20 × 10^4^, 1.69 × 10^5^, and 5.54 × 10^6^ cells/mL, respectively (Figure 2b).

### 2.3. Total Protein Expression Pattern of Breviolum

**Figure 3:**
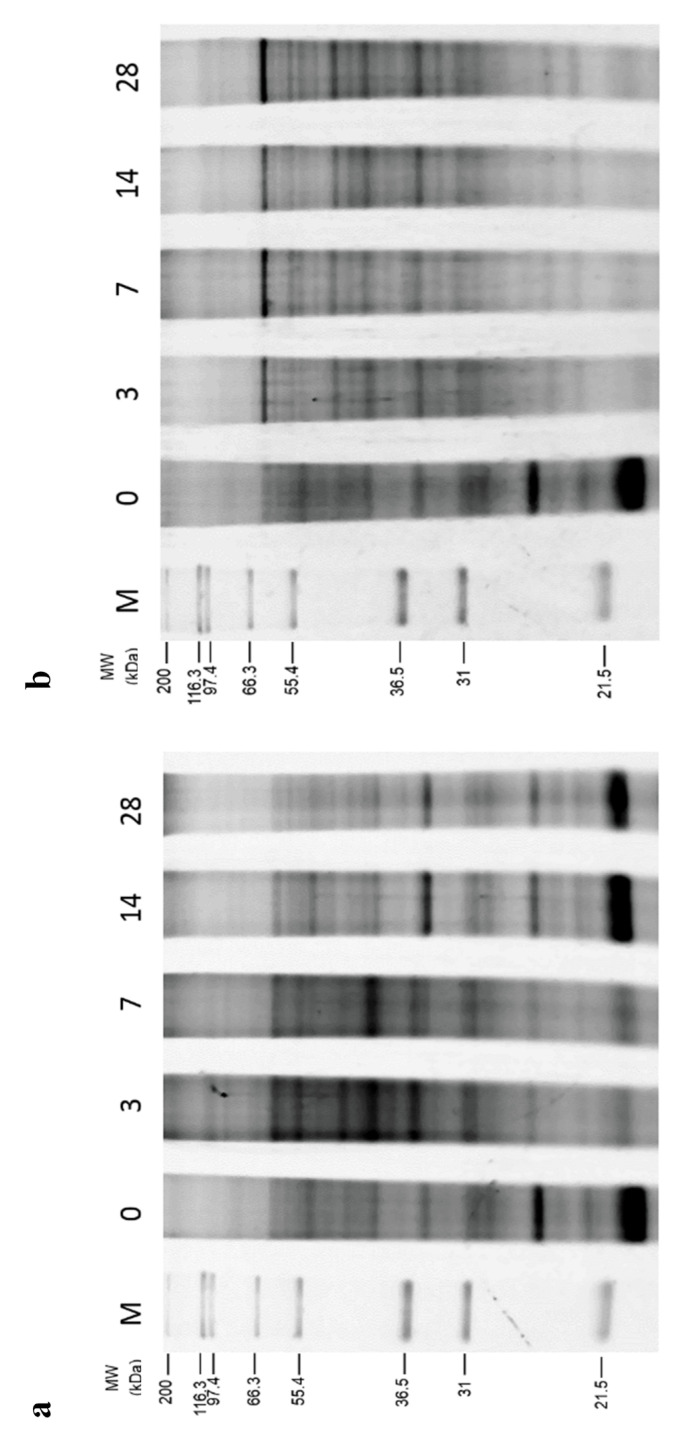
*Breviolum* protein expression for (**a**) MeOH and (**b**) PG treatment groups by the 3rd, 7th, 14th and 28th day of culture after thawing. The horizontal axis shows different days of culture, with M representing the marker and 0 representing *Breviolum* protein expression of untreated control group. The vertical axis indicates molecular size.

After extraction of *Breviolum* protein samples, an equal amount of total proteins were loaded in SDS-PAGE, and a SYPRO^®^ Ruby (Invitrogen, Heidelberg, Germany) staining gel confirmed that each protein sample was consistent for follow-up experimentation. Figure 3 showed that the total protein patterns were different for the MeOH (Figure 3a) and PG (Figure 3b) treatment groups of *Breviolum* undergoing two-step freezing. Results also showed that the total protein expression showed different patterns after 3, 7, 14, and 28 days of post-thawed culture.

### 2.4. LHP and RFP Protein Expression Pattern of Breviolum

**Figure 4:**
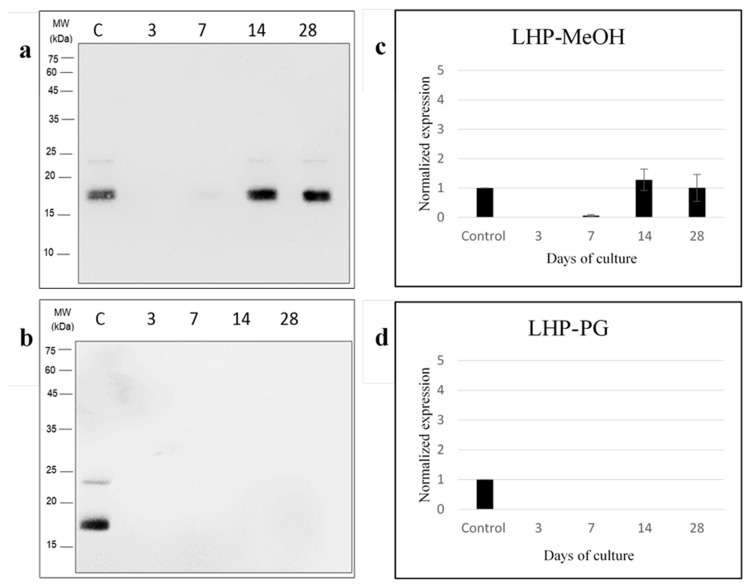
Western blot for *Breviolum* LHP expression for the (**a**) MeOH and (**b**) PG treatment groups after days of culture. The horizontal axis shows C as the control group (fresh *Breviolum* without freezing), and 3, 7, 14 and 28, as days of culture after thawing *Breviolum* LHP expression for the (**c**) MeOH and (**d**) PG treatment groups after same days of culture. The error bars represent standard deviation, and each group was repeated 3 times.

**Figure 5:**
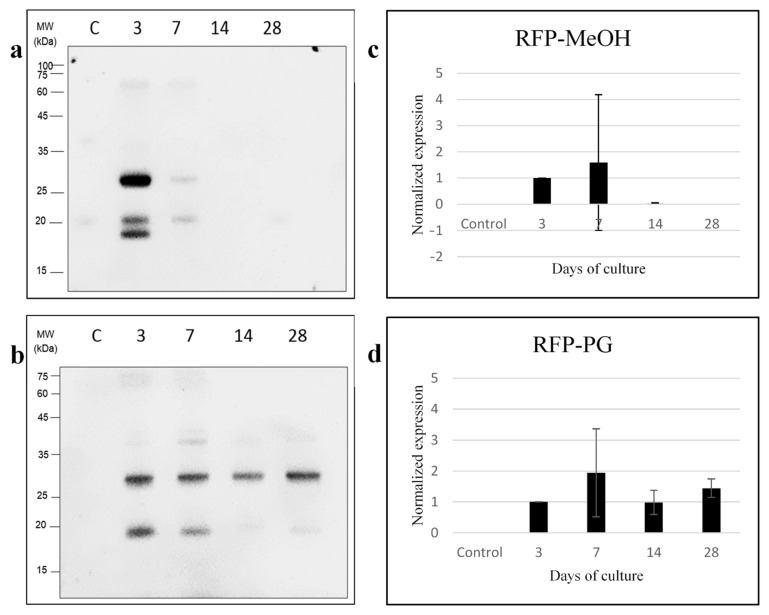
Western blot for *Breviolum* RFP expression for the (**a**) MeOH and (**b**) PG treatment groups after days of culture. The horizontal axis shows C as the control group (fresh *Breviolum* without freezing), and 3, 7, 14 and 28, as days of culture after thawing *Breviolum* RHP expression for the (**c**) MeOH and (**d**) PG treatment groups after same days of culture. The error bars represent standard deviation, and each group was repeated 3 times.

Western blotting was performed using anti-RFP and anti-LHP antibodies to detect the protein expression level of *Breviolum* at different time points during the post-thaw cultures in the MeOH and PG treatments (Figure 4 and Figure 5). For the MeOH treatment (Figure 4a,c), there was a significant effect of culture day on LHP expression (one-way ANOVA, F(4/10) = 15.311, *p* < 0.001). Specifically, no LHP was detected on the 3rd day of culture, though an LHP signal appeared on the 7th day, and expression peaked on the 14th. By the 28th day, its expression was not significantly different from the control group (post-hoc test *p* > 0.05). Meanwhile, for the PG treatment group (Figure 4b,d), LHP was never expressed over the full course of the 28-day culture. The effect of cryopreservation on RFP expression for both MeOH and PG treatment groups of *Breviolum* is shown in Figure 5. It was found that there was no expression of RFP from normal *Breviolum* culture, the control group. Meanwhile, for the MeOH treatment group (Figure 5a,c), the RFP expression was maximum on the 3rd day of culture but gradually reduced on the 7th day and could not be detected during 14–28 days. As for the PG treatment group (Figure 5b,d), RFP expression started right after thawing and continued over the whole course of the 28-day culture.

## 3. Discussion

Cryopreservation is now a common technique used among algae; however, studies of it regarding *Breviolium* and other dinoflagellates are still few [11,29,30]. There are many methods in algae cryopreservation, and the same is true for Symbiodiniaceae, including the two-step freezing [10,11,31] and the single-step direct immersion into liquid nitrogen at −196 °C [32,33]. In 2017, Zhao [10] applied vitrification, a rapid freezing method, to cryopreserve *Gerakladium*; the study found a much higher Symbiodiniaceae viability than programmable freezing that cools at a rate of 1 °C/min. Cooling rates vary among different freezing methods; in fact, it may change depending on the apparatus setting or procedure [8,9]. Our study chose *Breviolum* to freeze using the two-step cryopreservation with a cooling rate of 59.83 °C/min; the same cooling rate is also applicable to *Durusdinium* and *Gerakladium* [11,34]. In 2005, Gwo et al. [35] had attempted to freeze the algae *Nannochloropsis oculata* using the programmable freezing at a rate of 1 °C/min, down to different temperatures, for algae cryopreservation in liquid nitrogen; results found that by controlling the freezing rate, algal survival rates were higher than the single-step freezing. However, no research had yet been conducted on the single-step freezing, by direct liquid nitrogen immersion, for Symbiodiniaceae cryopreservation. Morris [36], in 1981, had mentioned that freezing involving single-step direct immersion of cells into liquid nitrogen did not provide the cell sufficient time to dehydrate and would thus lead to intracellular crystallization, injuring cellular membrane and organelles. On the contrary, if the cooling rate was overly slow, this may cause excessive dehydration, thus reducing the post-thawing recovery ability of the cell itself [12]. Hence, the cooling rate is an important factor in the freezing process. In the present study, the first stage of two-step freezing involved cooling in liquid nitrogen vapor at 59.83 °C/min. This alteration simplified the cooling method and made the overall setup handy, allowing greater mobility and convenience to the operation of cryopreservation.

The common choices of CPA for cryopreserving freshwater algae and marine algae, including dinoflagellates from the Symbiodiniaceae family, are permeating CPAs [12,31,34,36,37,38,39], for which DMSO is the most common type [13,32,37,40]. However, the present study showed that the DMSO treatment group during cryopreservation for *Breviolum* did not yield high *Breviolum* viability in comparison with the MeOH treatment group. A similar observation was found in the study of Chong et al. 2016c [11] with *Gerakladium*, in which MeOH added with sucrose gave rise to better viability than DMSO added with sucrose. In the 2007 study by Santiago-Vázquez et al. [12] on *Breviolum*, MeOH treatment also resulted in better viability after freezing than treatment with EtOH. Moreover, Hubálek 2003 [41] also showed that MeOH was the only CPA that successfully cryopreserved the cultured microalga *Euglena*; and in 2006, Rhodes et al. [42] successfully cryopreserved the microalgae *Chaetoceros calcitrans* and *Nitzcschia ovalis* using a low concentration (0.5%–5%) of MeOH. DMSO has been applied to the cryopreservation of many types of algae, but in this present study, DMSO did not yield high viability post-thaw. A possible reason for this is that while DMSO is non-toxic to cells at low temperatures (0–5 °C), its toxicity increases as temperature increased from room temperature [43]. Therefore, we propose that viability for the DMSO treatment group was reduced during equilibration at room temperature and during thawing due to toxicity of the cells, resulting in lower viability than that of the MeOH treatment group. Moreover, MeOH is more permeable than DMSO to cells, meaning MeOH enters cells and replaces intracellular water content at a faster rate, allowing the cells to reach osmotic equilibrium faster [43,44]. A faster equilibrium would be needed with the relatively fast cooling rate used in this study, compared to slower controlled rate cooling of <1 °C/min. This explains the higher viability for the MeOH treatment group than that of the DMSO treatment group when both underwent 20 min of equilibration. Although many studies have found suitable CPA for Symbiodiniaceae, the study of Hagedorn et al. 2010 [29] on *Cladocopium* found that the Symbiodiniaceae CPA tolerance did not change among different subclades within the genus. Our results have also shown that *Breviolum* from the 2 M MeOH treatment group showed higher ATP content than that of the control group. Our observations are in agreement with the studies by Chong et al. 2016c [11] and Lin et al. 2019 [34]. It was speculated that during the course of cryopreservation, Symbiodiniaceae lost its normal function and thus would require additional energy (such as ATP) for cellular repair.

In the present study, *Breviolum* was cultured for 3, 7, 14, and 28 days after cryopreservation. Results showed that *Breviolum* density began to increase at 2 weeks (14 days) after thawing. In 2007, Santiago-Vázquez et al. [12] conducted the first successful cryopreservation on *Breviolum*, isolated from corals, in liquid nitrogen using four different cryopreservation techniques. They pointed out that during culture, the cells first underwent a period of slow growth, then followed by a growth spurt; this can probably be explained by the fact that the cells need to adjust to their new environment. A similar growth trend was also observed in the present study even though the culture medium was kept the same prior to and after cryopreservation; the slow growth rate observed could also be explained by possible cryoinjuries in cells, causing the thawed *Breviolum* to prioritize cellular repair over growth.

The present study initially showed opposite trends in RFP and LHP expression between the cryopreserved symbionts and the control group in the first week of post-thaw culture. The subject of this study, *Breviolum*, is a temperature-sensitive organism. Based on the endosymbiotic relationship between Symbiodiniaceae and corals, it has been suggested that temperature change is often the main cause of disruption of their relationship [6]. The endosymbiotic relationship between Symbiodiniaceae and corals is compromised when the coral host and its endosymbionts are exposed to temperatures higher or lower than what they are acclimated to. Although relevant research regarding the effect of low temperature on algal proteins is still unavailable, similar research to the cryopreservation of fish oocytes and sperm had recorded protein denaturation [45,46]. In the study by Zilli et al. 2014 [47], it had been noted that many protein expressions from frozen-thawed fish sperm were lower than those of the control (non-treated sperm). Changes in protein expression within Symbiodiniaceae would affect its usual function, thus altering its endosymbiotic relationship with corals [48,49]. Hence, the investigation of protein content within Symbiodiniaceae would not only aid in the refinement of its cryopreservation but also in the understanding of the effect protein denaturation may have in the endosymbiosis with corals.

RFP, encoded in the genes, exhibits certain phenotypic trait in the organism that allows it to be used as a genetic marker in biotechnology [50]. RFP expression was associated with the oocyte formation in *Euphyllia ancora*, as it was expressed at the early stage of oogenesis and aided in breaking down hydrogen peroxide (H_2_O_2_), protecting the oocytes from oxidation [51]. RFP also has a protective function among corals; in Baird et al. 2009 [52] study, its expression increased when environmental conditions deteriorated, suggesting it to be some sort of protective mechanism. It is well documented that RFP is detected in coral host cells, but concentration within Symbiodiniaceae has not been observed. In our study, we found no detection of RFP in the control group, but RFP concentration peaked by the 7th day within cultured *Breviolum* in two CPA (MeOH and PG) treatment groups. This is the first documentation of RFP detection in Symbiodiniaceae. *Breviolum* within these two treatments showed higher growth compared to the control by the 14th day of our experiment (Figure 2). It is possible that the first 7 days were representative of the repair stage and that by the 14th day, the completion of cellular repair resulted in reduced RFP concentration. We propose that RFP may be an indicator of cellular stress or play a protective or repair role in Symbiodiniaceae; however, a robust understanding of the role of RFP in Symbiodiniaceae requires further investigation.

LHP is involved in Symbiodiniaceae photosynthesis; it was shown to be an internal control protein in Symbiodiniaceae [26]. Normally, Symbiodiniaceae should express a large amount of LHP in assistance to photosynthesis, as was observed in the control group *Breviolum* in the present study. The present study also found that LHP expression was affected after freezing; thus, LHP would not be a suitable internal control protein for cryotemperature experiments. After 28 days of culture in the PG treatment group, there was still no LHP expression, whereas by then, it was expressed in the MeOH group. The figure of cell growth for the PG treatment group showed that the *Breviolum* required a longer period of time to initiate cell growth; thus, it could be that LHP expression requires more time for recovery or that some cellular pathways were changed due to altered protein expression in the Symbiodiniaceae. Overall, PG is a more effective CPA than MeOH for cryopreservation of *Breviolum*. Meanwhile, for the MeOH treatment group, LHP expression started after 7 days of culture, meaning that *Breviolum* probably finished repair around the same time. LHP expression peaked by the 14th day, and by the 28th day, LHP expression showed no significant difference to that of the control group. The present authors had previously conducted cryopreservation of the dinoflagellate *Durusdinium* using 2 M PG as CPA [34], and the resulting analysis of LHP expression was similar to the present study. There was no LHP expression on the 3rd and 7th days of culture, and it was not until the 14th day that LHP expression was observed. Based on LHP expression, we suggest that the MeOH treatment group finished intracellular repair by the 28th day of culture, contrary to that of the PG treatment group, which did not finish its repair. Thus, the amount of LHP expression could be an indicator of Symbiodiniaceae repair after cryopreservation.

## 4. Materials and Methods

This study can be divided into two sections. The first section focused on the testing of different types and concentrations of CPAs and equilibration time for *Breviolum* cryopreservation by measuring their viability after 3, 7, 14, and 28 days of culture post-thawing. The second section made further analyses on the differing protein expression of frozen-thawed *Breviolum* over succeeding days of culture. The control group was not cryopreserved and monitored during the same period as the experimental Symbiodiniaceae. The *Breviolum* used in this study are the free-living algae isolated from the sea anemone *Exaiptasia diaphana* (previously named *Aiptasia pulchella*) [53].

### 4.1. Symbiodiniaceae Identification

Cladistic identification of Symbiodiniaceae was performed via the larger secondary sequence of the 23S rDNA from chloroplasts. Extraction of the Symbiodiniaceae DNA was performed using ZR Plant/Seed Kit (ZYMO Research, Irvine, CA, USA). Then, 10xAccuPrime PCR buffer solution was added together with 200 nM 23S rDNA primer and 5U/μL AccuPrime Pfx (Invitrogen, CA, USA) into a 50 uL sample. The sequence for the forward primer of the 23S rRNA is 5′-CACGACGTTGTAAAACGACGGCTGTAACTATAACGGTCC-3′, while the sequence for the reverse primer is 5′GGATAACAATTTCACACAGGCCATCGTATTGAACCCAGC3′ [54]. Polymerase chain reaction was performed for DNA amplification, first heating the sample to 94 °C for 5 min, for initial denaturation, then annealment occurred over two 30 s rounds at 94 and 50 °C, respectively, and finally, fragment elongation for 60 s under 72 °C. All steps were repeated for 30 cycles, and one final elongation at 72 °C for 10 min. Electrophoresis was performed for the product of PCR over 1% agarose gel, then dyed with ethidium bromide, followed by purification using Axygen DNA Gel Extraction Kit (Axygen Biosciences, Union City, CA, USA). The sample was then magnified and copied using PCR-Blunt II TOPO cloning kit (Invitrogen, Carlsbad, CA, USA), sequenced with ABI 3100 (Applied Biosystems, CA, USA), and finally, matched via BlastN for interspecies nucleotide sequence, confirming the specimens to belong to the genus *Breviolum*.

### 4.2. Culture of Breviolum

*Breviolum* was cultured in a plant culture chamber (LTI-613, Double Eagle, Taiwan), with temperature set to 25 °C, photoperiod of 12 h of light (40 μmol m^−2^ s^−1^), and 12 h of dark. The culture used autoclaved artificial seawater (A.S.W) (Coralife, scientific grade marine salt dissolved in deionized water, Franklin, WI, USA) together with f/2 (Guillard’s f/2 medium, Sigma-Aldrich, Saint Luis, MO, USA) added with penicillin (penicillin 10 units/mL, Sigma-Aldrich, USA). Thawed *Breviolum* was centrifuged (3000 rpm, 3 min, 25 °C), and the supernatant was removed. Then under the same setting, after adding the culture medium (for rinsing purposes), the sample was centrifuged again. Lastly, the sample was added into a T-75 cell culture flask (Nunc, Thermo Fisher Scientific, Waltham, MA, USA) for culture. Half of the culture medium was renewed each week to maintain the nutrient supply for *Breviolum*.

### 4.3. Preparation of Artificial Sea Water

First, 160 g of sea salt was dissolved into 5 L of pure water (Purelab Ultra, Elga, Antony, France); it was made sure that the salinity of the solution was 32%. This was followed by pouring 970 mL of seawater into each 1 L serum bottle, which was then autoclaved and cooled. Then, 30 mL of f/2 culture medium plus 1 mL of penicillin was added to the solution, mixed thoroughly, and filtered (0.45 μm Flow Filter, Thermo Scientific™ Nalgene™ Rapid-Flow™, IL, USA). Finally, the solution was stored in a refrigerator (Sampo, Kaohsiung, Taiwan) at 4 °C. The solution was warmed back to room temperature before each use.

### 4.4. Renewal of Culture Medium

Culture medium renewal had to be performed of the dark period during *Breviolum* culture. About 80 mL of the culture solution was poured out; the remaining was then gently swirled and mixed. The remaining medium was centrifuged in a 50 mL centrifuge flask, and the supernatant was removed. The medium was then returned to the serum bottle and filled back to 160 mL with fresh culture medium for continuing culture. After each renewal, 10 and 30 μL of the Symbiodiniaceae solution were removed for Symbiodiniaceae count by hemocytometer and for the adenosine triphosphate (ATP) bioassay, respectively.

### 4.5. ATP Bioassay

ATP bioassay was conducted prior to and after *Breviolum* cryopreservation to compare the change in *Breviolum* viability throughout the experiment. In normal cells, ATP acts as the energy currency for cellular functions, whereas its concentration reduces when the cell dies. Hence, ATP bioassays can be used as a preliminary assessment of cellular survival rate. This study performed ATP bioassays using ApoSENSOR™ Cell viability assay kit (BioVision, Milpitas, CA, USA). First, 5 μL of the sample was put into a luminescence test tube, followed by adding 100 μL of nucleotide releasing buffer, and was left for 3 min at room temperature for completion of the reaction. This was followed by adding 5 μL of ATP monitoring enzyme, thorough mixing and rest period another 30 s at room temperature. Finally, a measurement was made using a luminometer (Lumat LB 9507, Berthold Technologies GmbH & Co. KG, Germany). The use of nucleotide releasing buffer ruptured the *Breviolum*, allowing the binding of luciferin to ATP, catalyzed by luciferase, and the oxidation of ATP into adenosine monophosphate (AMP) and two phosphate groups, emitting a light blue light. The light was captured and measured by the luminometer for ATP concentration based on the light intensity.

### 4.6. Two-Step Freezing and Culture of Post-Thawed Breviolum

Three pools of the *Breviolum* for each treatment were analyzed, and the experiments were repeated three times. Therefore a total of nine measurements from three biological pools originating from treatment was obtained. The freezing method used for *Breviolum* in this study was the two-step freezing [11]. Prior to cryopreservation, the *Breviolum* were detached from the sides of the culture flask by gentle swirling and centrifugation (3000 rpm, 3 min, 25 °C) to remove excess culture medium. Then, *Breviolum* were separated by flushing the medium with a syringe (23 G × 1/2”, 0.45 × 13 mm, Top, Kaohsiung, Taiwan) 10 times. Then, the ATP bioassay was conducted after having confirmed with a hemocytometer that the *Breviolum* density was around 1 × 10^6^. After both measurements, centrifugation (3000 rpm, 3 min, 25 °C) was performed again to remove all remaining culture medium. Four cryoprotectants were tested, methanol (MeOH), propylene glycol (PG), dimethyl sulfoxide (DMSO), and glycerol (Gly) at concentrations of 1, 2, or 3 M. ASW was used as a base medium to dissolve the CPAs. CPAs were added directly onto the pelleted sample, which was resuspended by flushing with a syringe another ten times. Equilibration was performed at room temperature for 20 and 30 min for the CPA treatment groups. During equilibration, the sample was transferred into a 500 μL straw, and afterward, placed on the two-step freezing device (Taiwan patent M394447) [11], 5 cm above the surface of the liquid nitrogen (cooling rate at 59.83 °C/min) to cool for 30 min. After the first stage of cooling, the straw was immersed into liquid nitrogen for 2 h. Afterward, the sample was thawed in a 37 °C water bath for 10 s, followed by cell counts and an ATP bioassay analysis. Thawed *Breviolum* were cultured independently across 4 experiments with different culture durations (3, 7, 14, and 28 days). The number of algal cells was determined using a hemocytometer, and the relative cell culture density of *Breviolum* was normalized based on a culture duration-specific control group.

### 4.7. Protein Extraction

The best post-thaw results were obtained with the CPAs PG (2 M) and MeOH (2 M), and therefore only *Breviolum* that were cryopreserved with these two treatments were selected for the 3-, 7-, 14-, or 28-day cultures and for further experiments. Protein expression in each treatment was quantified three times. After culture, the *Breviolum* were transferred from the culture flask into a 50 mL centrifuge tube for centrifugation to remove excess culture medium. The pellet of *Breviolum* was transferred into a 1.5 mL centrifuge tube, and 500 μL of RIPA buffer [50 mM Tris-base pH 7.4, 0.5% NP-40, 0.125% Na-deoxycholate, 150 nM NaCl and 1 mM EDTA], 20 μL of protease inhibitor (Roche cat. no 11836153001), and micro glass beads (Sigma cat. no G9268) were added. The tube was then sealed with paraffin and placed into the TissueLyser LT (Qiagen Inc., Valencia, CA, USA) machine to homogenize the sample for 30 min at 25 Hz. Then the sample was centrifuged (15,000 rpm, 10 min, 4 °C), and the supernatant was transferred into a new 1.5 mL centrifuge tube. For protein precipitation, an equal volume of 20% trichloroacetic acid was added to the tube and then placed into a −20 °C freezer overnight. After protein precipitation, the solution was centrifuged (15,000 rpm, 10 min, 4 °C), and the supernatant was removed. The precipitated proteins were rinsed with acetone with 0.1% of dithiothreitol (DTT, 17-1318-02; GE Healthcare, USA) twice and then rinsed with pure acetone. Finally, the protein pellet was vacuum dried into a powder. The dry protein powder was stored in a −20 °C freezer and ready to be used.

### 4.8. SDS-PAGE and Western Blotting

The 1× SDS-PAGE sample buffer (62.5 mM Tris-HCl pH 6.8, 2% SDS, 10% glycerol, 10 mM DTT) was added to dissolve dry protein powder. Then, a Qubit^®^ Protein Assay Kit and a Qubit^®^ 2.0 fluorometer (Thermo Fisher Scientific, Waltham, MA, USA) were used to measure protein concentration. For the SDS-PAGE, 10 μg of each protein sample was separated by 14% polyacrylamide gel and stained with SYPRO^®^ Ruby protein dye (S12000, Invitrogen) according to the manufacturer’s instructions. The gel image was scanned using a laser scanning imager (Typhoon FLA 9500, GE Healthcare Life Sciences, Marlborough, MA, USA). For Western blotting, 5 μg of each protein sample were separated by 14% polyacrylamide gel and then blotted onto PVDF membranes (immobilon-PSQ 0.45 mm; Millipore, Darmstadt, Germany). The membranes were incubated in 5% skim milk/TBST buffer (Tris-buffered saline, 0.1% Tween-20) at RT for 1 h, followed by the incubation with the rabbit-anti-RFP antibody (1: 2500 dilution, cat. no ab59457, Abcam, Cambridge, UK), or mouse-anti-LHP antibody (1:10,000 dilution, custom antibody) in TBST buffer at 4 °C overnight. The membranes were then washed 3 times with TBST buffer for 5 min each time and incubated with anti-rabbit or anti-mouse IgG antibodies in TBST buffer for 1 h. The membranes were subsequently washed with TBST buffer and visualized using a SuperSignal West Pico Chemiluminescent substrate kit (cat. no 34080, Thermo Fisher Scientific) according to the manufacturer’s recommendations. Finally, the membranes were placed into a luminometer (Vilber Lourmat Fusion FX 7 Fluorescence/Luminescent CCD Image Analyzer, Vilber Lourmat, France), and an image was collected.

### 4.9. Protein Expression Analysis

Each experiment was repeated in triplicate to collect three images. Image J software was used for protein quantitative analysis. For each image, the position of the protein, based on its molecular size, was marked to show the relative position of each protein on the waveform plot. Then, the background value was zeroed to obtain the relative amount of protein concentration. Lastly, the 3 values from each treatment group were then averaged to obtain protein value. The relative amount was measured in each sample based on staining intensity.

### 4.10. Statistical Analysis

Statistical analysis for the data of the present study was conducted using the SPSS 17.0 version. Normality was verified with a Kolmogorov–Smirnov test, and homogeneity of variance was verified using Levene’s test. One-way analyses of variance (ANOVA) were conducted to detect equilibration time following different concentrations of CPA treatments. Tukey or Games–Howell post-hoc tests were used to verify differences between individual means, and *t*-tests were used to compare 20 vs. 30 min equilibration times at different CPA concentrations.

## 5. Conclusions

This study not only demonstrated that the protocols reported here do affect protein expression, but it also provided a suitable protocol for *Breviolum* cryopreservation. This is the first research conducted to investigate the effect of cryopreservation on Symbiodiniaceae protein expression as well as the first documentation of RFP expression in Symbiodiniaceae (RFP was an indicator of cell damage or cell stress). By gaining further understanding of the use of cryopreservation as a way to conserve Symbiodiniaceae, we hope to make an effort in the remediation and conservation of the coral reef ecosystem and provide additional methods to rescue coral reefs.

## Data Availability

Date is contained within the article.
